# Provider and community perceptions of integrated COVID-19 and routine childhood immunisation programmes in Nigeria: a qualitative exploratory study

**DOI:** 10.1186/s12913-024-11623-7

**Published:** 2024-10-21

**Authors:** Ayobami A. Bakare, Kofoworola O. Akinsola, Carina King, Abiodun A. Sogbesan, Oluwabunmi R. Bakare, Opeyemi Y. Fadahunsi, Julius Salako, Adegoke G. Falade, Sibylle Herzig van Wees

**Affiliations:** 1 Department of Global Public Health, Karolinska Institutet, Stockholm, Sweden; 2 Department of Community Medicine, University College Hospital, Ibadan, Nigeria; 3 Department of Paediatrics, University College Hospital, Ibadan, Nigeria; 4 Department of Paediatrics, College of Medicine, University of Ibadan, Ibadan, Nigeria

**Keywords:** Integrated vaccine delivery, Childhood immunisation, COVID-19 pandemic, Preventive care, Essential health services, Health systems, Nigeria

## Abstract

**Background:**

In Nigeria, COVID-19 vaccines were delivered through outreach activities, as well as integrated within routine immunisation. However, evaluations of integrated approaches for novel vaccines are scarce. We aimed to understand the perceived benefits and challenges of integrating the COVID-19 vaccine within routine immunisation in Nigeria, and identify ways to strengthen this approach.

**Methods:**

We conducted 30 semi-structured interviews with community members and healthcare workers in primary healthcare facilities (PHCs) in Jigawa (*n* = 16) and Oyo (*n* = 14) states, Nigeria from 08 August to 13 September 2022. Participants were selected purposively from PHCs. We obtained information on participants’ perception about routine immunisation, and perceived benefits and challenges associated with integrated COVID-19 vaccine delivery. Healthcare worker and community interviews were analysed separately following a thematic analysis approach.

**Results:**

We identified four themes that describe the community and healthcare workers’ responses, perceived impact, and the health system adaptions to the challenges associated with the integrated vaccine delivery approach. Community members expressed concern that children might be given COVID-19 vaccines instead of routine immunisations, while others appreciated the integrated approach due to their trust in the efficacy of COVID-19 vaccines, government, and healthcare providers. Healthcare providers perceived the integrated approach as improving vaccination coverage and awareness but noted additional problems of increased workload, vaccine scarcity, and prolonged clinic visits. Insufficient resources were subsisting barriers to effective integration in both states, but the provider’s gender was also a challenge in Jigawa state. Additionally, the use of incentives to generate demand had ambiguous effects in Jigawa state.

**Conclusion:**

Taking an integrated approach to deliver COVID-19 vaccines was acceptable by healthcare providers but community members expressed concerns. Given existing vaccination programmes have persistent challenges, it is pertinent to address these barriers to enhance effectiveness of an integrated approach.

**Supplementary Information:**

The online version contains supplementary material available at 10.1186/s12913-024-11623-7.

## Background

The COVID-19 pandemic led to significant negative impacts beyond mortality, including mental health issues, economic downturns, educational disruptions, increased unemployment, and put a strain on healthcare systems [1]. In an effort to effectively control the pandemic, over 13.1 billion doses of COVID-19 vaccines have been delivered to nearly all countries worldwide, with a primary dose coverage of 66% globally as of September 2023 [2]. In Africa, this number is lower, with 52% of the eligible population having been vaccinated [3]. In Nigeria, 77,285,627 eligible persons have completed the primary series of COVID-19 vaccination, equivalent to 37.7% of the population, by September 2023 and 7.5% receiving at least one booster dose [2]. This is far below the global target of vaccinating 75% of eligible populations in each country [2, 4, 5].

As of 5th June 2022, there were 256,250 confirmed cases, 3143 deaths and 3,137 active cases of COVID-19 infection according to the Nigeria Centre for Diseases Control (NCDC). Nigeria started COVID-19 vaccine roll-out in March 2021 [6, 7]. To improve COVID-19 vaccination coverage, the Federal Ministry of Health, through the National Primary Health Care Development Agency (NPHCDA) in Nigeria adopted the “whole family” approach in June 2022 [8, 9]. This approach integrates COVID-19 vaccination with other basic primary healthcare services, such as childhood vaccination, and screenings for hypertension, diabetes and malnutrition, with the aim of vaccinating all eligible populations (everyone aged 18 years and older) in Nigeria [10]. Several other health programmes have previously been integrated into the primary health care service delivery in this way, including vitamin A supplementation in children aged 6–59 months, intermittent preventive treatment for malaria prevention in infants and pregnant mothers (IPTi and IPTp), and HIV testing and treatment. However, these integrations have come with associated challenges, including insufficient human resources, inadequate finances, and increased workload for healthcare providers [11].

In Nigeria, little is known about the impact of integrating COVID-19 vaccination into existing routine immunisation programmes. A study conducted in two primary healthcare facilities in Abuja reported that the “whole family” approach increased utilization of priority health services such as COVID-19 and routine immunizations [12]. However, the study was a quasi-experimental study implemented to test the approach [12]. As a consequence of the decline in childhood vaccination coverage due to the COVID-19 pandemic, concerns have emerged regarding resilience of existing vaccination services [13, 14]. This underscores the necessity for intensive efforts to address this decline through an integrated vaccination approach [8]. It also highlights a need to understand reasons for this decline through research.

Assessing providers’ and community perceptions of integrated COVID-19 and routine immunisation programmes in Nigeria is crucial to understand the perceived benefits and challenges of integrating these public health interventions and identify ways to strengthen the approach. We therefore aimed to understand the perceived benefits and challenges of integrating the COVID-19 vaccine within routine immunization in Nigeria This evidence will support the Nigerian government, policymakers, stakeholders in the Ministry of Health, and other healthcare providers with valuable insights into how to strengthen integrated vaccination programmes in the health system, particularly during times of public health emergencies like the COVID-19 pandemic.

## Methods

### Study design

This is an exploratory qualitative study using reflective thematic analysis according to Braun and Clark [15]. We conducted semi-structured interviews between 08 August and 13 September 2022 with community members and healthcare providers in primary health care facilities (PHCs) in Jigawa and Oyo states, Nigeria. Semi-structured interviews allowed for discussions with healthcare workers and community members to understand their perceptions of the benefits and challenges associated with integrated COVID-19 vaccine delivery. We followed the Consolidated Criteria for Reporting Qualitative Research in the presenting our findings [16].

### Setting

Oyo State is located in Southwest region, while Jigawa state is in the Northwest region of Nigeria. The study was conducted in two local government areas (LGAs) in each state: Egbeda and Ibadan Southwest LGAs in Oyo state, and Dutse and Kiyawa LGAs in Jigawa state.

In 2022, Oyo state had a projected population of 7,976,100, with 23.3% COVID-19 vaccine coverage [4, 17]. Healthcare in this state is delivered through both public and private sectors. The most common occupations in Oyo state include agriculture, small and medium scale entrepreneurship, and civil service. In addition, 9.8% of the population lives in severe poverty, while 70% of its population resides in the highest wealth quintile. The mean international wealth index for Oyo state is 51.4%, with a literacy rate of 50.8% and 52.4% for women and men, respectively [18, 19]. In 2021 the under-five mortality rate was estimated to be 57 deaths per 1000 live births. However, there is still under-performance in immunisation coverage as 55.8% of children aged 12–23 months having not received all the basic vaccines, and 24.9 receiving no vaccinations [19].

In Jigawa, the estimated total population is 7,499,100 and 66.7% of the population had received COVID-19 vaccination as of June 2023 [2, 20]. Jigawa is predominantly rural, driven by an agrarian economy, and over 80% of the population working as farmers. The under-five mortality rate in Jigawa is 174 deaths per 1000 live births and 56.8% of children aged 12–23 months had not received all the basic vaccines, while 42.6%% had no vaccinations [19]. Jigawa has the highest proportion of people belonging to the lowest wealth quintile, with 57.5% of its population living in severe poverty. The mean international wealth index for Jigawa state is 28.5% and the literacy level is 6.7% and 26.7% for women and men, respectively [19].

### Participants and sampling

We purposively selected two Local Government Areas (LGAs) in each state - Ibadan-Southwest (urban) and Lagelu (peri-urban) in Oyo, and Dutse (urban) and Kiyawa (rural) in Jigawa. We included LGAs with urban and rural characteristics considering these may influence programme implementation and participants’ perceptions. We also considered feasibility of data collection and availability of structures and resources for leveraging of community entry and stakeholder’s engagement.

We purposively recruited 14 health care providers (*n* = 8 Jigawa, and *n* = 6 Oyo) who were involved in routine childhood and COVID-19 immunisation delivery at primary health care level. The healthcare workers included a nurse (*n* = 1), health educators (*n* = 1), community health workers (*n* = 10), and an immunisation monitoring and evaluation officer (*n* = 1). We also interviewed the local government immunisation officer and cold chain officer at the LGA level to ensure representation of various cadres and different roles in immunisation service delivery at primary health care level. We recruited 16 community members (*n* = 8 Jigawa, and *n* = 8 Oyo) visiting the PHC facilities for routine childhood and COVID-19 immunisation, clinic consultations, and other health services utilization using convenience sampling (Table 1). This sample size was determined based on practical considerations of the time needed to recruit participants and expectation that the number would be sufficient to achieve saturation. It is further in line with findings from a systematic review that showed that saturation can be reached with 9–17 semi-structured interviews [21].

**Table 1:** Socio-demographic characteristics of participants

Participants characteristics	Community Members		Healthcare providers	
	Oyo (n=8)	Jigawa (n=8)	Oyo (n=6)	Jigawa (n=8)
Sex				
Male	2	1	0	6
Female	6	7	6	2
Religion				
Islam	5	8	3	8
Christianity	3	0	3	0
Level of education				
Primary/no formal	0	4	0	0
Secondary	2	3	1	0
Tertiary	6	0	5	8

### Data collection

The research team comprised public health specialists. In Jigawa, data collection was completed by experienced research assistants who are fluent in English and Hausa languages and have knowledge of the context. In Oyo, interviews were conducted by a research assistant with experience in qualitative research and knowledge of the local context. The interviewers lived in Oyo and Jigawa, during and after the COVID-19 pandemic [22, 23]. None of the data collectors were related to the study participants. Semi-structured interviews were conducted in locations conducive and appropriate for the participants and interviewers, which sometimes included home visits and workplaces for community members. Interviews with healthcare workers took place at healthcare facilities. All interviews were audio-recorded, translated and transcribed verbatim into English for analysis. Data was stored in a secure cloud platform with access granted to research team members only. Interviews in Jigawa were conducted in English and Hausa, and interviews in Oyo were conducted in English and Yoruba languages. Field notes were made during the interviews and no repeat interviews were carried out.

### Data analysis

After cross-checking the transcripts, healthcare worker and community data were analysed separately using thematic analysis [15]. The data analysis team conducted a data-driven thematic analysis to develop themes and sub-themes as summarized in Table 2. The data were reviewed separately, with coding performed for health care workers’ interviews and then the coding of community members’ interviews by the data analysis team independently. Themes were created then refined in an iterative process by the analysis team until consensus was reached.

**Table 2:** Results from analysis

Themes	Codes
Perceived benefits of an integrated approach	• Community appreciation of the integrated approach • Increased community access to health information • Efficiency in health service delivery • Improvement in vaccination coverage
Community hesitation towards integrated vaccination programme	• Fear of children being given COVID 19 vaccine reduced attendance at routine immunisation clinics • Gender preference for vaccinator • Lack of trust in government • Misinformation
Healthcare workers overburdened with integrated approach	• Work overload for healthcare workers • Shortage of antigens for routine immunisation
Health system adaptions required for integrated immunisation services	• Stipend for healthcare workers • Training of healthcare workers • Ad-hoc recruitment of staff • Use of mobile team for monitoring • Rescheduling vaccination appointment due to vaccine stock out • Community mobilization and counselling • Use of information, education, and counselling (IEC) materials

### Reflexivity

AAB is a community health physician who has knowledge of local context and has previously investigated COVID-19 vaccine perception at roll-out in similar setting in Nigeria. SvHW is a social scientist with extensive research experience on vaccine hesitancy. KOA and OYF are female researchers with at least bachelor’s degree and has understanding of local context.

### Trustworthiness

We interviewed various categories of participants to capture different perceptions. All interviews were conducted in the language the participants preferred. We triangulated our findings among various categories of participants. In addition, data analysis was done by three people who had different backgrounds and thus brought in different perspectives. Findings were also discussed with the wider team who had contextual knowledge.

### Findings

Data analysis revealed four themes - Table 2. There were overlaps in the findings between the two settings, and in cases where there were variances, we explicitly state them below.

### Perceived benefits of an integrated approach

Healthcare providers believed that the integrated approach promotes efficient service delivery, and it is effective in improving COVID-19 and routine immunisation vaccination coverage and overall health awareness .

In both Oyo and Jigawa state, we found that community members and health care workers were appreciative of the integrated vaccination programme as it provides an opportunity for free health checks including blood pressure and sugar levels.

*I love it (the integrated approach) because it’s an avenue for people to also know the status of their health. *Caregiver 02 Oyo

*During routine immunisation*,* we use the opportunity to educate people on COVID-19 immunisation and its benefits. We tell nursing mothers(caregivers) that they can now take COVID-19 vaccine*,* and also use that opportunity to give them the COVID-19 vaccine*. HCW 02 Jigawa.

Healthcare workers further highlighted that they perceived a benefit for more generalised vaccination coverage and more efficient use of limited resources:

*We now have a lot of people taking the routine immunisation. Before we have many measles children that didn’t come for the routine immunisation but presently with the COVID-19 immunisation people are coming from far place for routine immunisation.* HCW 01 Oyo.

### Community hesitation towards integrated vaccination programme

Despite some positive views toward the integrated programme, we found community members also expressed their uncertainty towards integrated delivery of COVID-19 vaccination and routine immunisation. In Jigawa, a vaccine mandate by the government, in part, led to doubt and fear among community members that their children may be given COVID-19 vaccines together with routine immunisations. Healthcare workers noted that this led to a noticeable decline in clinic attendance for routine immunisation of children.

*A lot of parents stopped bringing their children for child’s immunisation*,* because they feel the way the government is making it mandatory for all*,* they may start giving children the vaccine [COVID-19] together with their routine immunisation. So that made some parents to stop taking their children for immunisation.* Caregiver 02 Jigawa.

Moreover, in Jigawa, healthcare workers consistently emphasised the community’s preference for a specific gender of healthcare workers when getting vaccinated, as a contributory factor to community hesitation towards vaccination.

*Another challenge we have is that some men in some communities do not allow us to immunize because we are female health workers. They prefer men to men and female to female. Also*,* if the vaccinators are all men*,* the female will not receive the COVID-19 vaccines.* HCW 05 Jigawa.

Distrust in the government and health system emerged as a recurrent topic among interviewees in Jigawa. Community members cited controversies around vaccine production such as politicization of the COVID-19 vaccine because of incentives, perceived secrecy, and lack of transparency in vaccine production as reasons for this distrust. This distrust also manifested in caregiver’s perceptions of the integrated approach as a bait to get them immunised with the COVID-19 vaccine.

*Parents were afraid then [due to incentivisation] because of the thought of using their babies as a bait to immunize them against COVID-19.* Caregiver 05 Jigawa.

*This COVID-19 of a thing*,* it is not everybody that has accepted it. Some people have the belief that it is just pack of lies and not enough honesty about the vaccine. COVID-19 vaccine is just a way to enrich them (a way for certain people to make money)*. Caregiver 05 Oyo.

Misinformation about vaccines in general was another reason why community members hesitated towards the integrated vaccination programme. This played a substantial role in influencing the perception and attitude of community members towards vaccine uptake.

*They usually object (routine immunization for their children)*,* for instance in the case of polio they will say it’s acid. Another said her child will not be immunized because he will grow up behaving like the Jews. Some don’t even allow their children to be marked using the marker because they suspect that the marker contains the drug.* Caregiver 01 Jigawa.

Many interviewees mentioned exposure to COVID-19 vaccine myths and conspiracy theories through social media platforms and community narratives, which were believed to cause widespread anxiety and doubt about the COVID-19 vaccine, and the integrated vaccination approach within the community.

*Some refuse the COVID-19 vaccine because they taught it is for family planning in the community*,* some are saying the government brought the disease and some are saying is to reduce the number of people in Nigeria because we are many. Everybody with his/her taught (narrative) about the COVID-19 vaccine*. Caregiver 03 Jigawa.

Thus, existing myths and misinformation about both childhood and the COVID-19 vaccine arguably affected views and concerns about an integrated approach.

### Healthcare workers overburdened with integrated approach

Although healthcare providers acknowledged that the integrated approach promotes efficient service delivery, and it is effective in improving COVID-19 vaccination coverage and awareness, the integrated approach created additional problems of work overload for healthcare providers and scarcity of vaccines for routine immunisation.

W*e are short of staff. For example*,* I met two people in the facility and 20 people wanted to collect vaccines. So*,* they had to share (the work): one would be giving COVID-19 (vaccine)*,* the other would give RI [routine immunisation].* HCW 05 Oyo.

In Oyo state, scarcity of routine antigens was highlighted as a result of the integrated vaccination approach, thereby becoming an additional source of burden to the healthcare workers, as they have to call the caregivers when the vaccines become available. Healthcare workers mentioned that there had been low availability of routine antigens and administration to children due to the focus on COVID-19 vaccine distribution and administration.

*It’s affecting us on the supply of RI antigens*,* they are really concentrating on the COVID-19 vaccine. Because before we didn’t have problems with availability of RI antigens but during this COVID-19 period there was scarcity of some RI vaccines. There will be no month that there won’t be scarcity of one RI or the other. And this is affecting our record because we won’t be able to vaccinate the children at the right time. When it’s now available we may call 10 and only 2 will come back.* HCW 01 OYO.

This shows that healthcare workers expressed concerns of an integrated approach for the success of wider vaccination efforts.

### Health system adaptions for integrated immunisation services

This theme describes perceptions on strategies employed to counter some challenges identified in the integrated delivery of COVID-19 and routine immunisation programmes. Strategies included COVID-19 training of healthcare workers, ad-hoc recruitment of staff for vaccination, outreach and monitoring, provision of stipends for healthcare providers as transport support, demand generation for routine immunisation and COVID-19 vaccines.

*Stipends are given to work more. It ought to cover transportation from maybe the vaccination stand to their houses. When we started last year*,* the fee was ₦1000 per day but presently it’s ₦2000 per day*. *The strategy that’s also working well is having more people on our team. Adding more team members has been very helpful. Our outreach and mobile teams are helping us reach everyone who needs to be vaccinated.* HCW 01 OYO.

Efforts to create demand for COVID-19 and routine vaccines within an integrated approach were made through creating awareness, providing incentives for community members, particularly in Jigawa, counselling, trained healthcare providers, community mobilization using the available information, education, and counselling (IEC) materials.

*We used IEC materials- posters and banners to give people information and for sensitization although there’s supposed to be one banner per team and we have 10 and it’s not enough*. HCW 01 OYO.

Whilst healthcare workers appreciated the strategies put in place to support integrated immunisation services, there were concerns at the level of the community.

In Jigawa, incentives for caregivers reduced the hesitancy against the routine immunisation programme. However, it did not improve community knowledge about the importance of vaccines generally, as healthcare workers highlighted the incentives led to community members rejecting vaccination during outreach programme or expressing demand for a vaccine with a higher associated incentive. There were no reports of incentives for vaccine recipients either for routine or COVID-19 immunization in Oyo state.

*Coming to the hospital now has rapidly increased than before due to the incentive. So even if you go to homes or communities (for community outreach)*,* they will just tell you to go that they will come to the hospital for their vaccination. They will promise you to come to the hospital rather than receiving the vaccination at home* HCW 04 Jigawa.

*Most of the times*,* mothers are lazy to take their children for immunization*,* the money being given entices many women. A lot of women start taking their kids for immunization not because of the importance but because of the money.* Caregiver 05 Jigawa.

Similarly, some caregivers highlighted that some of them deliberately delayed or missed vaccination for their children because they were waiting for the fifth vaccination visit that comes with a greater sum of financial incentive (₦2,000/$2.5). Interestingly incentivisation for COVID-19 vaccination led to politicization of the COVID-19 vaccination programme, feeling of suspicion by community members and inequitable access.

*I noticed some women came at 9 months of birth and had never had immunization for their children*, *but because at 9months*, *they will be given ₦2000*, *so they brought the children.* Caregiver 02 Jigawa.

*So*,* there was a time we suggested from the LGA here that we want to boost our COVID-19 vaccine coverage by vaccinating more eligible people. Since we know there were people that are receiving money ranging from 5*,*000 to 20*,*000 monthly from the Buhari money (president intervention fund)*,* so we used the opportunity to get them. We made a law that no COVID-19 vaccine no collection of money. At time we got a lot of people*,* unfortunately for us they stopped the money sharing programme*,* so some people refused to accept the second dose. Some even refused to accept a dose saying until that Buhari money sharing resumes before they would accept the vaccine. HCW 06 Jigawa*.

## Discussion

We aimed to understand healthcare providers’ and community members’ perceptions of benefits and challenges of integrated COVID-19 and routine immunisation programmes in two states in Nigeria. Our findings reveal that the integrated approach has many benefits, but community members showed hesitation towards it for various reasons. Healthcare workers also expressed concerns with this approach, in relation to availability of antigens for routine immunisations and the added workload it resulted in for them. The use of incentives demonstrated ambiguous effects on routine immunisation uptake among community members.

Our study revealed that the integrated approach to healthcare delivery was seen to have some significant benefits, particularly in the improving COVID-19 and routine immunization vaccination coverage, as well as overall health monitoring and awareness. Healthcare providers noted the approach’s efficiency in service delivery, highlighting its effectiveness in increasing vaccination coverage and promoting general health status among communities. It emphasises the holistic advantage of the integrated approach in enhancing community health outcomes.

Nevertheless, some community members showed hesitation towards the integrated vaccination programme. This was primarily driven by the fear that children might be vaccinated with a COVID-19 antigen. This fear could stem from distrust in the government and health workers and/or misinformation. This resonates with findings elsewhere globally, and in Africa, suggesting that distrust in government, health providers and vaccines can instil fear, leading to reluctance towards the integrated approach [24]. Similarly, a study conducted in Lagos, Nigeria, found general distrust in the government’s motives regarding the vaccine and uncertainties about its future effects [25, 26]. This underscores the urgent need for proactive efforts by the government and health providers to foster trust and disseminate information through community sensitisation, active involvement in health-related policies and programmes, as pre-emptive measure to combat infodemic, improve vaccine acceptability [27], and to strengthen the linkage between community and health facilities for the effective integration of vaccination services.

A notable factor contributing to community hesitation in Jigawa was the preference for healthcare providers based on gender – since adults were now also receiving vaccines as opposed to only their children where gender was not such a concern. Healthcare workers expressed concerns about community’s distinct preference for female HCWs to administer vaccines to women and girls, emphasising their desire for more female immunisation service providers in line with socio-cultural norms. A similar finding was reported in another study conducted in Northwest Nigeria [28]. These socio-cultural norms are made more apparent in an integrated approach because people of all ages and genders receive the vaccination, as opposed to infants only. This could result in delayed or missed immunisation for children due to the inevitable unavailability of female healthcare workers as a result of insufficient human resource for health in this setting [29, 30]. To address this preference and potential barriers to timely immunisation, active involvement of the male gender in health education sessions and community sensitisations is essential, empahsizing the benefits of vaccines and the implications of poor vaccination uptake on disease control. Moreover, increasing the number of female healthcare workers, especially in culturally sensitive regions, can address gender-based preferences and improve vaccine uptake.

Our study revealed that incentivisation of health programmes can be problematic, and thus it requires broad consideration and continuous evaluation. While provision of incentives in Jigawa reduced hesitancy towards the routine immunisation programme, it presented challenges of incomplete immunisation. The lack of apparent benefits of financial incentives on vaccine uptake in our study agrees with previous studies which also found limited or inconclusive benefits of such interventions [31, 32]. In Jigawa state, prioritisation of vaccine with higher financial incentives points to caregiver’s lack of understanding of benefits of complete immunisation, and may also be as a result of poverty given that 85% of the population are in the poorest quintile [31, 33]. These findings suggest the need for holistic approach to tackle underlying causes of vaccine hesitancy, particularly improving vaccine knowledge and addressing vaccine misinformation and concerns of hesitant people.

Furthermore, our study found that integrating COVID-19 vaccination into existing immunisation programmes placed an additional burden on healthcare workers, resulting in both work overload and a shortage of vaccines for routine immunisation. This may be attributed to more attention given to COVID-19 vaccination logistics at the expense of the routine immunisation programme, or it could be due to a shortage of materials and human resources for the effective delivery of both programmes. The World Health Organization and the United Nations Children’s Fund (UNICEF) similarly highlight in their document on the considerations for integrating COVID-19 vaccination into immunisation programmes and primary health care, the possibility of human resources becoming overstretched, and health workers and support staff becoming overloaded and fatigued [34, 35]. This indicates the necessity to strengthen health system’s capacity to effectively manage integration services. In addition, continuous and inclusive stakeholder engagement is important to address unintended effects and enhance effectiveness and sustainability of integrated programmes.

Finally, strengthening the healthcare workforce and resources is essential to manage the additional burden of integrated programmes and prevent overloading healthcare workers. This includes continuing and expanding existing strategies such as the use of mobile teams, outreach programmes, community sensitization, and regular monitoring and evaluation. Additionally, leveraging the integrated approach to offer routine health checks can enhance overall health awareness. Engaging stakeholders, including community leaders and policymakers, in the development and implementation of these programmes is vital for ensuring cultural sensitivity and contextual appropriateness to improve the effectiveness and acceptance of integrated vaccination efforts.

### Limitations

The key limitation in our study was that perceptions were gathered only from community members and healthcare providers who were present at primary health centres. This excluded both those receiving vaccinations in private facilities, and perceptions from community members who do not engage with the health system. Despite this limitation, our findings provide context-specific insights into integrated COVID-19 and routine immunisation programmes in Nigeria and may inform future public health responses and approach to improve vaccine uptake. Findings from our study may be transferrable to other low and middle-income countries with a similar weak health system and sociocultural norms.

## Conclusion

The integrated vaccination approach was generally acceptable to healthcare workers but was met with some hesitation from the community, and there were complaints of work overloads and insufficient logistic support from healthcare workers. Hence, there is a need for enhanced strategies to deliver this programme. Addressing challenges related to vaccine hesitancy, incentivisation, and healthcare worker burden is critical for successful and sustainable integration. Future research should expand its focus to include secondary and tertiary public and private health facilities for a more comprehensive understanding.

## Supplementary Information

Supplementary Material 1.

## Data Availability

The datasets used and/or analysed during the current study are available from the corresponding author on reasonable request.
