# Supplementary material for: Provider and community perceptions of integrated COVID-19 and routine childhood immunisation programmes in Nigeria: a qualitative exploratory study
Source: BMC Health Serv Res. 2024 Oct 21;24:1262. doi: 10.1186/s12913-024-11623-7 (PMC11492757; doi:10.1186/s12913-024-11623-7)
Supplement: Supplementary file 1 — Supplementary Material 1. [file 12913_2024_11623_MOESM1_ESM.docx]

**ANNEXES**

**Appendix I**

Healthcare provider interview guide

We will be conducting interviews with healthcare providers who work in primary health facilities that offer routine immunisation and COVID-19 immunisation services

1. Clinical context

Can you tell me about a typical immunisation clinic (routine immunisation) in your setting?

- Probe: What sort of activities are done? How many children do you see on
- average?
- Probe frequency of clinics; clinic duration o Probe preparation done prior to the clinic

- If the facility provides COVID-19 immunisation services:

- Probe: What sort of activities are done? What role does the participant?
- perform? How many recipients do you attend to on average per day?
- Probe frequency of clinics, clinic hours.
- Probe preparation done prior to the clinic (COVID-19 vaccination).
- If not, probe why it does not offer COVID-19 immunisation services?
- What are the processes involved to receive COVID-19 vaccine?
- What is working well with COVID-19 immunisation delivery in Nigeria?
- What is not working well with COVID-19 immunisation delivery in Nigeria?

1. Perception of integrated COVID-19/routine immunisation deliveries
2. What is your opinion of providing COVID-19 immunisation with other routine immunisation services in your facility as recommended by the NPHCDA?
3. Does it increase coverage for COVID-19 vaccine? How?
4. How do healthcare providers consider that approach? Does it help your work? How?
5. NPHCDA also recommends screening for hypertension and diabetes alongside COVID-19 vaccination. To what extent this is implemented at your facility?
6. How is this approached perceived by the community members.
7. Existing challenges with routine immunisation services
8. What is working well with routine immunisation delivery in Nigeria?
9. What is not working well with routine immunisation delivery in Nigeria?
10. Impact of COVID-19 immunisation services on routine immunisation service
11. In what ways has COVID-19 immunisation programme affected routine immunisation delivery in your facility?
    1. Probe positive impacts if any
    2. Probe negative impact if any
    3. Has COVID-19 affected people’s perception of vaccines generally in Nigeria? How?
    4. What effects does routine immunisation have on COVID-19 immunisation programme?
    5. Tell me more, can you give examples?
12. COVID-19 vaccine recipients
13. Have you received at least a dose of the COVID-19 vaccine before? If yes your experience (registration, verification, and actual inoculation)
14. Probe motivation for acceptability or hesitancy
15. Confidence in vaccine
16. Complacency
17. Convenience
18. Comment on the steps involved to get vaccinated. Were the steps necessary? Why?
19. Experiences post-vaccination, side effects if any? How were they managed?
20. Were the side effects expected? How?
21. Perceived difference between COVID-19 immunisation services and RI
22. Challenges encountered
23. Why do some people receive the COVID-19 vaccine?
24. Why are other people not willing to accept the vaccine?
25. Do you have any other thing to tell me?
26. Thank you for your time

**Appendix II**

**Community members interview guide**

We will be conducting interviews with non-healthcare providers who present in primary health facilities.

1. Getting to know the participants

- Tell me about yourself: where you live, work you do, marital status, religion and tribe

1. Tell me your perception of immunisation generally? Why?
2. What vaccine(s) have you received in your adulthood? Why?
3. Do you take your children for immunisation? Why?
4. Many under-five children are not fully immunized in Nigeria. What could be responsible for this?
5. Any other challenges regarding immunisation programme for children in Nigeria?
6. What is working well with the ways routine immunisation for children is delivered in Nigeria?
7. What is not working well with the children’s routine immunisation?
8. COVID-19 vaccination experience
9. Have you received at least a dose of the COVID-19 vaccine before? If yes, when and how many doses have you received? Share your experience (registration, verification, and actual inoculation), and how satisfied were you with the entire process?
   1. Probe motivation for acceptability or hesitancy
   2. Why do some people receive the COVID-19 vaccine?
   3. Why are other people not willing to accept the vaccine?
   4. Comment on the steps involved to get vaccinated. Were the steps necessary? Why?
   5. What were your experiences post-vaccination, side effects if any? How were they managed? Were the side effects expected? How?
   6. What is not working well with COVID-19 immunisation delivery in Nigeria?
   7. What do you consider to be working well with COVID-19 immunisation in Nigeria?
10. Impact of COVID-19 immunisation services on routine immunisation service

a. Of the challenges you discussed so far, which ones are peculiar to:

1. Routine immunisation? How
2. COVID-19 vaccine programme? How?
3. Based on your opinion and experiences, in what ways has the COVID-19 immunisation programme affected immunisation for children in your setting?
4. Probe negative impact if any
5. Probe positive impacts if any
6. Has COVID-19 immunisation programme shaped how people perceive vaccines generally?
7. We have talked about effects of COVID-19 vaccine on routine immunisation. Now what effects does routine immunisation have on COVID-19 immunisation programme?
8. Tell me more, can you give examples?
9. The federal government has launched “whole family approach” to COVID-19 vaccination, such that you can bring your child for routine immunisation while you yourself can take COVID-19 vaccine and get screened for other disease like hypertension, diabetes.
10. What is your impression about this? Do you think it will encourage more people to receive COVID-19 vaccine or otherwise?
11. Do you have any other things to tell me?
12. Thank you for your time
